# The mitochondrial unfolded protein response and mitohormesis: a perspective on metabolic diseases

**DOI:** 10.1530/JME-18-0005

**Published:** 2018-05-30

**Authors:** Hyon-Seung Yi, Joon Young Chang, Minho Shong

**Affiliations:** 1 Research Center for Endocrine and Metabolic DiseasesChungnam National University School of Medicine, Daejeon, Korea; 2 Department of Medical ScienceChungnam National University School of Medicine, Daejeon, Korea

**Keywords:** mitochondria, oxidative phosphorylation, mitochondrial unfolded protein response, diabetes, insulin resistance

## Abstract

Mitochondria perform essential roles as crucial organelles for cellular and systemic energy homeostasis, and as signaling hubs, which coordinate nuclear transcriptional responses to the intra- and extra-cellular environment. Complex human diseases, including diabetes, obesity, fatty liver disease and aging-related degenerative diseases are associated with alterations in mitochondrial oxidative phosphorylation (OxPhos) function. However, a recent series of studies in animal models have revealed that an integrated response to tolerable mitochondrial stress appears to render cells less susceptible to subsequent aging processes and metabolic stresses, which is a key feature of mitohormesis. The mitochondrial unfolded protein response (UPR^mt^) is a central part of the mitohormetic response and is a retrograde signaling pathway, which utilizes the mitochondria-to-nucleus communication network. Our understanding of the UPR^mt^ has contributed to elucidating the role of mitochondria in metabolic adaptation and lifespan regulation. In this review, we discuss and integrate recent data from the literature on the present status of mitochondrial OxPhos function in the development of metabolic diseases, relying on evidence from human and other animal studies, which points to alterations in mitochondrial function as a key factor in the regulation of metabolic diseases and conclude with a discussion on the specific roles of UPR^mt^ and mitohormesis as a novel therapeutic strategy for the treatment of obesity and insulin resistance.

## Introduction

Mitochondria are double membrane-bound organelles that resemble α-proteobacteria, from which they are thought to have originated by endocytosis more than 1 billion years ago (Gray* et al*. 2001). As the powerhouses of the cell, mitochondria generate most of the energy through oxidative phosphorylation (OxPhos), a process in which electrons are passed along a series of OxPhos subunit proteins embedded in the inner mitochondrial membrane (Huttemann* et al*. 2007). Apart from cellular respiration and energy synthesis, mitochondria are also required for the metabolism of nucleotides as well as biosynthesis of amino acids and lipids.

**Figure 1:**
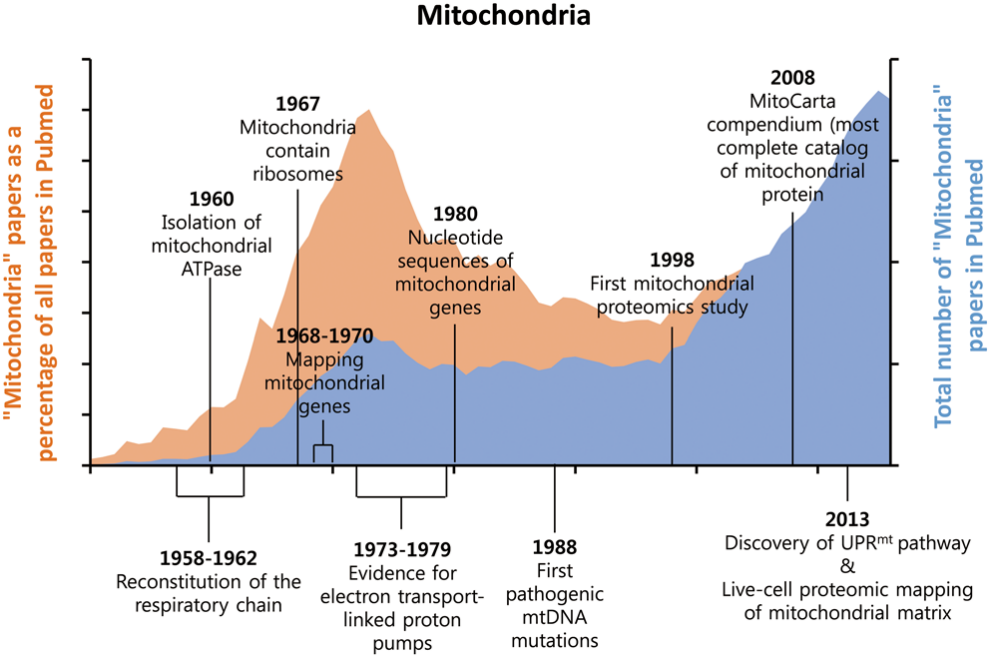
Scientific interests in mitochondria from 1950 to 2016. The articles were identified in PubMed using the term ‘mitochondria’ for each year from 1950 to 2016. The articles were expressed as the total number of mitochondria articles out of all articles, as well as mitochondria articles as a percentage of all articles in PubMed. The essential milestones and discoveries in the field of mitochondrial research are described by the year of publication.

**Figure 2:**
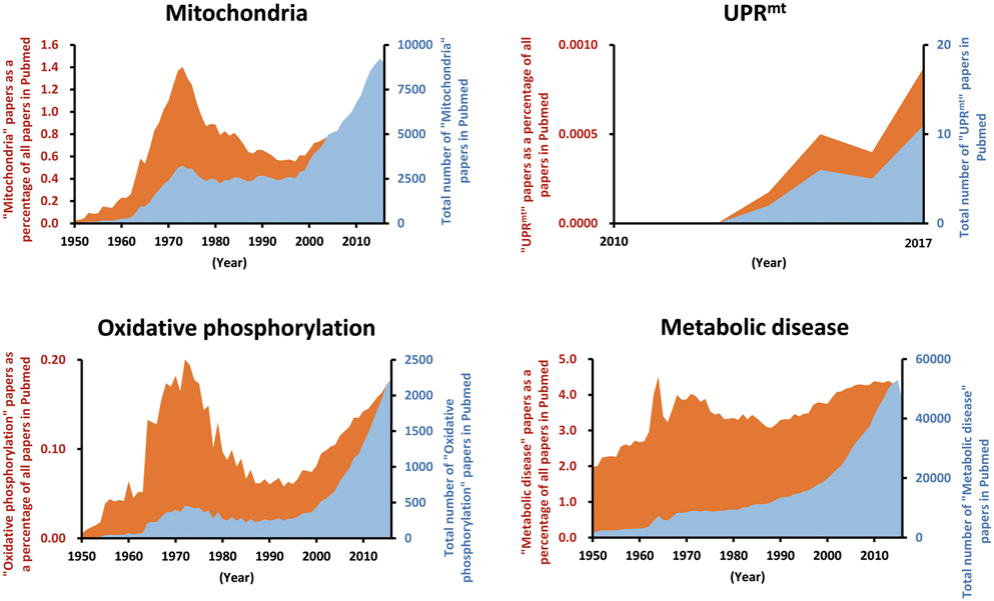
Total number and percentage of ‘Mitochondria,’ ‘Oxidative phosphorylation,’ ‘UPR^mt^’ and ‘Metabolic disease’ articles in PubMed from 1950 to 2016.

The discovery in the early 1960s that mitochondria have their own DNA and translation system stimulated an increase in articles related to mitochondria and oxidative phosphorylation from the late 1960s to 1980 (Figs 1 and 2). Mitochondrial ATPase was initially purified from beef heart mitochondria and its action as a coupling factor was also demonstrated (Penefsky* et al*. 1960, Pullman* et al*. 1960). Moreover, the positional papers regarding the evidences for electron transport-linked proton pumps and oxidative phosphorylation were published in the 1970s (Figs 1 and 2) (Guerrieri & Nelson 1975, Brand* et al*. 1976, Wikstrom & Krab 1979). These intensive works on mitochondrial OxPhos complex contributed to launching the golden era of mitochondrial research from the 1960s to late 1970s.

Recently, a second renaissance in mitochondrial biology is emerging due to the recognition of the mitochondria as a central regulator in metabolic homeostasis and longevity. In 1998, 46 mitochondrial proteins were reported by 2D gel electrophoresis of purified human placental mitochondria, and the mitochondrial proteins were also identified by peptide mass fingerprinting (Fig. 1) (Rabilloud* et al*. 1998). Five years later, in 2003, state-of-the art mass spectrometry-based proteomics enabled us to discover more mitochondrial proteins in yeast, mice and humans (Mootha* et al*. 2003*a*, Sickmann* et al*. 2003, Taylor* et al*. 2003). These data have been consolidated in the MitoCarta, an inventory of mammalian mitochondrial genes and provides integrated datasets of mitochondrial protein localizations in various tissues through mass spectrometry and large-scale GFP tagging/microscopy (Pagliarini* et al*. 2008). In addition, a series of recent studies revealed the complete structure of the mammalian respiratory chain complex and mitochondrial ribosomes by single-particle cryoelectron microscopy (Brown* et al*. 2014, Gu* et al*. 2016, Englmeier* et al*. 2017). Thus, new technologies for discovery of uncharacterized mitochondrial proteins are promoting the development of mitochondrial biology (Fig. 1) (Kazak* et al*. 2013, Rhee* et al*. 2013). To date, the successful efforts to understand the mitochondrial OxPhos complex and to catalog the mitochondrial proteome have established the relevance of mitochondrial dysfunction in a broad spectrum of metabolic diseases.

By far, one of the most significant discoveries during this second renaissance has been the identification of the mitochondrial unfolded protein response (UPR^mt^), a retrograde stress response induced by mitochondrial proteotoxic stress. The UPR^mt^ was discovered in the late 1990s and is an area of increased scientific interest for research on aging and metabolic diseases (Figs 1 and 2) (Martinus* et al*. 1996, Ryan* et al*. 1997). Although initially characterized in mammalian cells, mechanistic insight on the UPR^mt^ was gleaned from studies in worms and flies. Recently, the mechanism of UPR^mt^ regulation was partially revealed in mammals as well as worms (Haynes* et al*. 2010, Nargund* et al*. 2015, Chung* et al*. 2017*b*). Thus, many researchers are trying to define the role of UPR^mt^ in mammalian cells and tissues, and its possible role in mitohormesis. The mitochondrial chaperones and proteases predicted to be involved in the regulation of mitochondrial proteostasis may have an essential role in the modulation of metabolic diseases in mammalian cells and mice. While many review articles focused on the role of UPR^mt^ in lower organisms, a review focusing on the effects of UPR^mt^ on metabolic diseases are scarce in mammals.

In this review, we discuss the recent remarkable progress in understanding the role of mitochondrial OxPhos perturbations on systemic energy metabolism that may be relevant in the treatment of obesity and diabetes. We also describe the effects of UPR^mt^ on cellular and systemic physiology during the conditions of mitochondrial dysfunction and proteotoxicity. Lastly, we discuss the UPR^mt^ and mitohormesis as new therapeutic targets for treatment of metabolic diseases.

## OxPhos function in a variety of metabolic diseases

OxPhos, as a main platform for energy production in eukaryotic cells, consists of two major components including the phosphorylation of ADP to ATP using energy from the chemiosmotic proton gradient, as well as the oxidation of molecules generated during the breakdown of glucose. Five multimeric complexes embedded in the inner mitochondrial membrane comprise the OxPhos system. Complexes I-IV are multi-subunit enzymes, which work to generate an electrochemical proton gradient across the mitochondrial inner membrane, which is used by the complex V (ATP synthase) to produce ATP (Chaban* et al*. 2014). Thus, dysfunction of the mitochondrial OxPhos system not only is correlated with mitochondrial genetic disorders but also complex human diseases such as metabolic and neurodegenerative diseases. Therefore, understanding the systems and functions of OxPhos allows us to unravel not only the significance of mitochondrial respiratory chains but also the novel therapeutic options in the etiology and progression of metabolic diseases.

### OxPhos function and metabolic diseases

Metabolic diseases including type 2 diabetes mellitus (T2DM) and obesity are closely associated with the alteration of gene expression involved in OxPhos. The skeletal muscle of patients with T2DM showed a decrease in expression of a global set of genes of the OxPhos pathway (Sreekumar* et al*. 2002, Mootha* et al*. 2003*b*). High-fat diet decreases the expression of the genes necessary for OxPhos, mitochondrial proteins and mitochondrial biogenesis in humans and mice (Sparks* et al*. 2005). The rate of mitochondrial OxPhos activity in skeletal muscle were 30% lower in insulin-resistant subjects compared to control subjects (Petersen* et al*. 2004). Additionally, the intramyocellular triglyceride content in the soleus muscle was 80% higher in the subjects with insulin resistance (Petersen *et al*. 2004). Impairment in OxPhos function induced by deficiency of *Crif1*, an integral mitoribosomal factor for the insertion of nascent OxPhos polypeptides into the inner mitochondrial membrane, in beta cells triggers progressive failure of insulin secretion and cellular proliferation (Kim* et al*. 2015). Beta-cell-specific knockout of *Tfam*, a transcription factor of mtDNA-encoded OxPhos polypeptides, promotes hyperglycemia by impaired secretion of insulin from islets in response to glucose stimulation (Silva* et al*. 2000). Haploinsufficiency of pentatricopeptide repeat domain 1 protein, known as a regulator of mitochondrial gene expression, reduces mitochondrial respiratory complex biogenesis and function, thus resulting in obesity and insulin resistance (Perks* et al*. 2017). These data suggest that impaired OxPhos has been associated with the development of insulin resistance, the decrease of insulin secretion from beta cells, and the dysregulation of fatty acid metabolism in mice and humans.

However, multiple lines of evidences indicate that there may not be a causal relation in all circumstances. For example, Asian Indians with insulin resistance have similar skeletal muscle mitochondrial OxPhos capacity as nondiabetic controls. In addition, regardless of diabetic status, Indians have higher OxPhos capacity than Northern European Americans, indicating that mitochondrial OxPhos dysfunction cannot account for all insulin resistance in Asian Indians (Nair* et al*. 2008). Further, mitochondrial OxPhos capacity can be altered by fuel intake, oxidative load, epigenetics and environmental factors (Patti & Corvera 2010). Therefore, whether impaired mitochondrial oxidative activity causes insulin resistance or results from insulin resistance is not certain and requires further research.

It is well established that reactive oxygen species (ROS) is an important mediator of metabolic dysfunction (Rani* et al*. 2016). Mitochondria are the primary site of ROS production, most notably due to premature leakage of electrons from complexes I and III of the electron transport chain. Thus, it is unsurprising that reduction of mitochondrial ROS leads to an improved metabolic phenotype. The Mediterranean diet showing health-promoting effects reduces the expression of OxPhos genes, thereby leading to reduction of ROS in peripheral blood mononuclear cells (van Dijk* et al*. 2012). Mitochondria-derived ROS is also associated with massive macrophage accumulation in adipose tissue, leading to exacerbation of insulin resistance and systemic inflammation (Han 2016). Several studies showed that caloric restriction, which induces protection from aging and metabolic diseases, prevents the decline in mitochondrial respiratory function (Hempenstall* et al*. 2012, Lanza* et al*. 2012). However, caloric restriction also decreases the expression of the genes involved in OxPhos and oxidative stress in peripheral blood mononuclear cells and skeletal muscle (Kayo* et al*. 2001, Crujeiras* et al*. 2008, Lanza *et al*. 2012). In addition, intracellular ROS produced by OxPhos pathway is associated with hyperglycemia and is important in the pathogenesis of diabetic nephropathy (Huang* et al*. 2006). Muscle- or liver-specific deletion of mitochondrial flavoprotein apoptosis inducing factor results in OxPhos deficiency, but leads to the improvement of glucose tolerance and insulin sensitivity in mice (Pospisilik* et al*. 2007).

Thus, the relationship between mitochondrial OxPhos function and metabolic diseases is highly complicated and is also likely to be context dependent. Although mitochondrial OxPhos capacity has been considered as a key factor underlying aging and metabolic diseases, the attenuation of OxPhos function may be a useful therapeutic strategy for obesity and insulin resistance. More in-depth mechanistic insight can be gained from studies on the OxPhos quality control systems.

### The role of mitochondrial chaperone systems in metabolism

**Figure 3:**
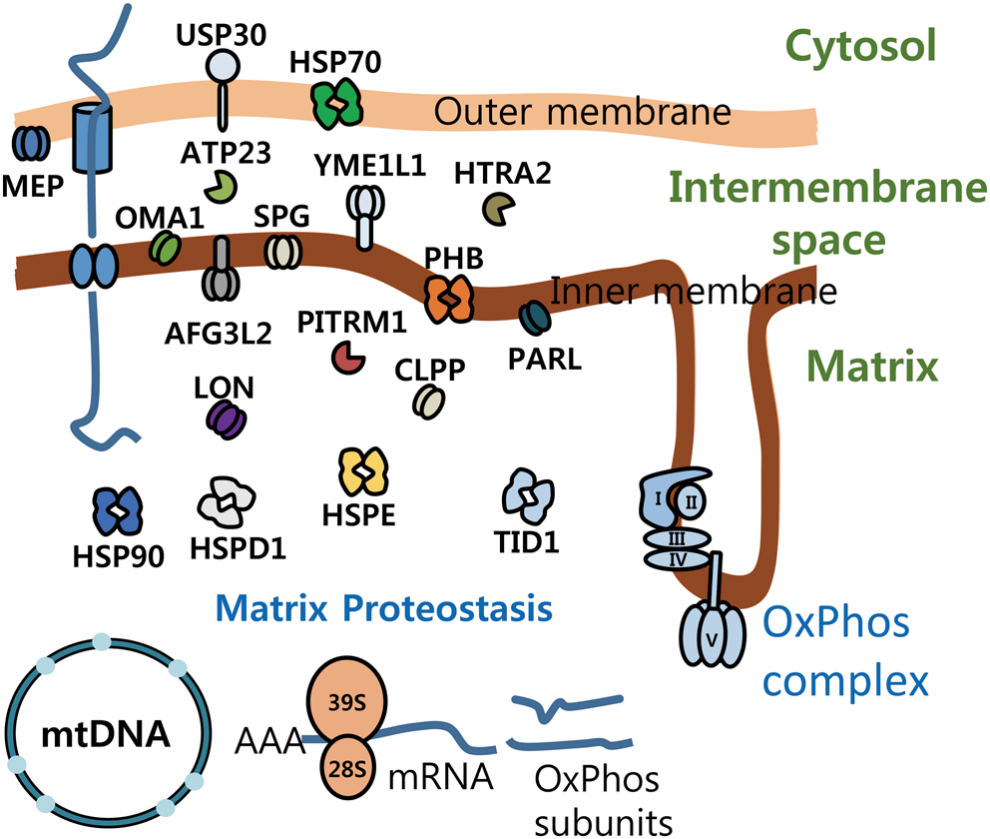
Mitochondrial chaperones and proteases. Schematic cartoon describes the major factors involved in mitochondrial proteostasis. The chaperones and proteases are located in mitochondrial outer and inner membrane, intermembrane space and matrix.

As the majority of mitochondrial proteins are encoded by the nuclear genome and imported into the mitochondria in an unfolded state, mitochondrial chaperones play an important role in maintaining normal mitochondrial function. In conditions of increased mitochondrial proteotoxic stress, the mitochondrial unfolded protein response (UPR^mt^) is activated and upregulates a specific set of genes, of which the mitochondrial chaperone system is involved (Aldridge* et al*. 2007). In this context, the mitochondrial chaperone systems are required for facilitating protein-folding within the mitochondria (Fig. 3). While HSP60 plays a prominent role in the UPR^mt^ response, other mitochondrial chaperones are yet to be fully explored in this context.

The heat shock protein 60 (HSP60) has been studied extensively and is established as a marker of the UPR^mt^ induction in lower organisms. HSP60 is reduced in the hypothalamus of obese or diabetic mice and humans, which is associated with central insulin resistance and mitochondrial dysfunction. The role of leptin on mitochondrial function and insulin sensitivity in hypothalamus is dependent on mitochondrial HSP60, suggesting that the hypothalamic mitochondrial chaperone system has an important effect on systemic energy homeostasis in obesity and metabolic diseases (Kleinridders* et al*. 2013). Moreover, heat shock protein family D member 1 (Hspd1) knockdown decreased mitochondrial respiration, levels of respiratory subunits and mtDNA levels in the murine hypothalamic cell line (Kleinridders *et al*. 2013). Furthermore, myocardial HSPD1 increases in high-fructose-fed rats, which is associated with prevention of hyperglycemia and severe cardiac injury (Chen* et al*. 2009). siRNA-induced *Hspd1* knockdown also increased intracellular protein aggregation and oxidized proteins in renal tubular cells, which may contribute to diabetes-induced renal tubular dysfunction (Aluksanasuwan* et al*. 2017). Thus, abnormal regulation of HSP60 may decrease the capacity of mitochondrial function and the UPR^mt^ response, which potentially links the UPR^mt^ to metabolic disorders. However, more extensive studies would be required, especially in mammalian systems, to firmly establish a causative role for UPR^mt^ dysregulation in the etiology and progression of diseases.

Several studies have revealed that the mitochondrial HSP90s play a key role in organelle homeostasis such as protein-folding, quality control, redox balance and the regulation of metabolic pathways (Chae* et al*. 2013). The HSP90 chaperone machinery in association with HSP70 is also involved in protein-folding and activation of newly synthesized proinflammatory signal transducers (Caplan* et al*. 2007). HSP90 inhibition induced by 17-dimethylaminoethylamino-17-demethoxygeldanamycin attenuates inflammatory signaling pathways including NF-kB, STAT1 and STAT3 in diabetic kidneys, thus resulting in amelioration of diabetic nephropathy and atherosclerosis by the induction of protective HSP70 (Lazaro* et al*. 2015).

TNFR-associated protein 1 (TRAP1), which belongs to the HSP90 family, is also involved in the regulation of mitochondrial respiration and glycolysis. Loss of TRAP1 leads to a reduction in oxidative damage and cell-cycle defects by global reprogramming of cellular bioenergetics, leading to decreases in aging-related pathologies including obesity and tumor formation (Lisanti* et al*. 2014).

Prohibitin, one of the evolutionarily conserved mitochondrial chaperones, has been implicated in adipose tissue biology from worms to mice (Merkwirth & Langer 2009). Overexpression of prohibitin in adipocytes leads to an increase in mitochondrial biogenesis and obesity development (Ande* et al*. 2014). However, the metabolic phenotypes of obesity induced by overexpression of prohibitin in adipocytes are sex specific due to the prohibitin translocation from mitochondria to the nucleus in response to estrogen (Dong* et al*. 2013). Overexpression of prohibitin in adipocytes impaired glucose homeostasis in male mice, but female mice did not show any changes in the glucose tolerance test (Ande *et al*. 2014). This suggests that mitochondrial chaperones do not always result in the improvement of metabolic phenotype in mice.

Other studies suggest that HSP72 is closely associated with insulin resistance and type 2 diabetes. Muscle tissues of patients with type 2 diabetes exhibit lower expression of *HSP72* compared to those of normal controls (Kurucz* et al*. 2002). Intramuscular *Hsp72* mRNA expression is inversely correlated with insulin-stimulated glucose disposal rate during a hyperinsulinemic–euglycemic clamp in patients with type 2 diabetes (Bruce* et al*. 2003). Overexpression of *Hsp72* in skeletal muscle increases mitochondrial number and OxPhos function, leading to enhanced energy expenditure and insulin sensitivity in mice (Henstridge* et al*. 2014). In contrast, *Hsp72*-deficient mice exhibit obesity and insulin resistance, suggesting that HSP72 enhances muscle insulin sensitivity by promoting fatty acid oxidation and reducing fat storage and adiposity in skeletal muscle via the HSP72-Parkin axis (Drew* et al*. 2014).

Although it is unclear how mitochondrial chaperones contribute to the improvement of obesity and insulin resistance, recent works indicate a role in the recovery of mitochondrial function and biogenesis in mice (Ande *et al*. 2014, Bottinger* et al*. 2015). However, it should be noted that while the mitochondrial chaperone-mediated regulation of metabolic homeostasis is established in worms and mice, less is known regarding the regulation of mitochondrial proteostasis in humans. To date, it is unknown as to which key factors regulate the UPR^mt^ in mammals. Additionally, knockout models of specific mitochondrial chaperones and proteases in mammals did not reveal any abnormal mitochondrial proteostasis. Therefore, many studies have just shown the transcript levels of mitochondrial chaperones and proteases as markers of UPR^mt^ induction *in vitro* and *in vivo* (Aldridge *et al*. 2007, Haynes *et al*. 2010, Fiorese* et al*. 2016). Recent studies demonstrated that the ATF4 pathway is activated in mammals upon mitochondrial stress and mediates the mitochondrial stress response (Quiros* et al*. 2017). However, mitochondrial stress caused by LONP1 knockdown or mdivi-1 exposure in mammalian cells does not induce prototypical UPR^mt^, as defined by the induction of HSP60, HSP10 and CLPP. This suggests that mitochondrial stress-mediated activation of the ATF4 pathway is not always involved in the regulation of mitochondrial chaperones and proteases in mammalian cells.

### Role of intrinsic mitochondrial proteases in metabolism

In addition to the chaperone system, mitochondria also contain evolutionarily conserved mitochondrial proteases as crucial elements of mitochondrial quality control (Fig. 3). The inner mitochondrial membrane protease OMA1 has essential roles in mitochondrial quality control and metabolic homeostasis. Deficiency of* Oma1* leads to increased body weight and fat contents, as well as impaired thermogenesis after diet-induced obesity in mice. *Oma1*-deficient mice displayed improved glucose intolerance and increased insulin sensitivity on a normal chow diet. However, *Oma1*-deficient mice lost these metabolic benefits in the condition of high-fat diet-induced obesity (Quiros* et al*. 2012).

The LON protease (also LONP1) is an important enzyme in the degradation of oxidized proteins within the mitochondrial matrix, which is highly induced in response to acute stress. A decline in LON levels is associated with aging and chronic stress, thereby promoting the development of aging-related diseases by losing the ability to induce LON during acute stress (Ngo* et al*. 2011). LON is also involved in the regulation of hepatic insulin resistance and gluconeogenesis. Treatment with *LON*-specific siRNA induces mitochondrial dysfunction such as reduction in cellular ATP contents and mitochondrial membrane potential. Deficiency of LON protease also causes hepatic gluconeogenesis by induction of glucose-6-phosphatase and PGC-1a in human liver SK-HEP-1 cells. Overexpression of LON protease ameliorates hepatic insulin resistance in the cells containing dysfunctional mitochondria (Lee* et al*. 2011). These data suggest that mitochondrial proteases contribute to the improvement of systemic energy metabolism via regulation of mitochondrial function and quality control.

CLPP, another matrix protease, plays a crucial role in the UPR^mt^. Loss of CLPP causes moderate mitochondrial respiratory deficiency by defective mitoribosome assembly and a decrease in mitochondrial translation rates (Szczepanowska* et al*. 2016). Depletion of *Clpp* ameliorates cardiomyopathy in heart-specific DARS2-deficient mice via increased mitochondrial OxPhos function (Seiferling* et al*. 2016). In addition, the expression of CLPP and LON, which are major mitochondrial proteases, are used as an indicator of UPR^mt^ activation *in vivo* as well as *in vitro* (Houtkooper* et al*. 2013, Munch & Harper 2016). ATP-dependent metalloprotease *Yme1l* deficiency in cardiomyocytes causes dilated cardiomyopathy and heart failure by activation of OPA1-mediated mitochondrial fragmentation. Cardiac function and mitochondrial dynamics were restored by Oma1 ablation in cardiomyocyte-specific *Yme1l*-deficient mice (Wai* et al*. 2015).

Mitochondrial protease presenilin-associated rhomboid-like (PARL) also contributes to the regulation of skeletal muscle OxPhos and insulin signaling. PARL mRNA expression in skeletal muscle is reduced in elderly subjects and patients with type 2 diabetes. The expression of *PARL* in gastrocnemius muscle is correlated with insulin sensitivity as assessed by whole-body glucose disposal during a hyperinsulinemic–euglycemic clamp. Moreover, Leu262Val in *PARL* is associated with increased plasma insulin levels in human subjects (Walder* et al*. 2005). Muscle-specific knockdown of PARL results in lower mitochondrial energetics and impaired insulin signaling as well as malformation of mitochondrial cristae and increased oxidative stress in mice (Civitarese* et al*. 2010). This suggests that *PARL* is an important candidate gene for the development of insulin resistance and type 2 diabetes. Taken together, a network of conserved proteases distributed across mitochondrial compartments may not only regulate the onset and progression of obesity and insulin resistance, but also play pivotal roles in the maintenance of mitochondrial function.

## UPR^mt^ and mitohormesis in organismal models

In contrast to linear models of mitochondrial dysfunction and disease progression, studies in the past decade have established a new field of research with important ramifications for the field of longevity and aging-related diseases, including metabolic diseases. Although mitochondrial perturbation generally leads to metabolic dysfunction, mild mitochondrial perturbation in yeast showed increased replicative lifespan through a retrograde stress response from the mitochondria to the nucleus (Kirchman* et al*. 1999). This was corroborated in studies of *Caenorhabditis elegans* and *Drosophila*, where mild mitochondrial perturbation led to increased lifespan in both models (Dillin* et al*. 2002, Owusu-Ansah* et al*. 2013). This concept has come to be defined as mitochondrial hormesis or mitohormesis, where mild mitochondrial perturbation increases fitness and confers a resistance to subsequent stresses (Yun & Finkel 2014). Although various pathways have been reported to be involved in the mitohormetic process, one area of interest is the UPR^mt^, which will be explored in more detail in the following sections.

### The mitochondrial unfolded protein response (UPR^mt^)

Due to the endosymbiotic origin from α-proteobacteria, mitochondria contain their own genome, the mtDNA, which only encodes ~1% of the total mitochondrial proteome including 13 OxPhos proteins, 22 transfer RNAs and 2 ribosomal RNAs (Calvo & Mootha 2010). Multiple factors such as absence of protective histone molecules and proximity of the mtDNA to the inner mitochondrial membrane, where ROS are generated, contribute to a higher mutation rate in mtDNA, thereby promoting mitochondrial proteotoxicity and a decline in mitochondrial function (Krishnan & Turnbull 2010, Cha* et al*. 2015). In this context, the concerted efforts of the mitochondrial system of proteases and chaperones are pivotal in maintaining protein homeostasis or proteostasis within the mitochondria.

Altered mitochondrial proteostasis and dysfunction triggers the activation of a retrograde signaling pathway from mitochondria to the nucleus that results in the upregulation of mitochondrial chaperones and proteases to re-establish mitochondrial function. This response is known as the UPR^mt^. The UPR^mt^ was first characterized in mammalian COS7 cells, where the presence of mutated ornithine transcarbomylase (deltOT) within the mitochondrial matrix resulted in the mitochondrial-specific upregulation of Hsp60 and Clpp, in the absence of endoplasmic reticulum stress (Zhao* et al*. 2002). Despite its origins in mammalian cells, mechanistic insight was gained from studies in *C. elegans*. Most notably, the master regulator of the UPR^mt^ in worms was revealed to be Atfs1 and reliant on the co-factors DVE1 and UBL5 (Haynes* et al*. 2007, 2010). A subsequent study by the same group found that the UPR^mt^ in worms is regulated by two complementary pathways; an Atfs1-dependent pathway and ROS-mediated pathway dependent on the phosphorylation of the GCN2, a eukaryotic initiation factor 2 alpha (eif2α) kinase (Baker* et al*. 2012). This suggests that the UPR^mt^ may be regulated in a similar fashion to the UPR^er^, where temporary translation inhibition may be accompanied by upregulation of chaperones and proteases to re-establish organellular homeostasis. Moreover, the reliance on ROS may suggest that there may be an interconnection between the UPR^mt^ and mitohormetic pathways, as low levels of ROS have been implicated in improved cellular fitness and lifespan extension in various animal models (Lapointe & Hekimi 2008, Yang & Hekimi 2010, Owusu-Ansah *et al*. 2013).

Recent efforts to define this process in mammalian models suggests that the UPR^mt^ response may be differentially regulated in higher organisms, and likely reliant on an integrated stress response (Melber & Haynes 2018). Due to previous studies which have established Hsp60 and Clpp as important factors upregulated during the UPR^mt^, the current gold standard in mammals is the detection of these markers under conditions of mitochondrial perturbation. However, it should be noted that indiscriminate use of these markers may lead to inaccurate conclusions as the UPR^mt^ may be regulated differently in mammalian models. Nevertheless, the UPR^mt^ is essential for the repair and recovery of the mitochondrial protein network, leading to proper cellular function.

### The UPR^mt^ as a mitohormetic response

The concept of UPR^mt^ and mitohormesis has been explored previously in the context of longevity studies in worms and flies. Mitohormesis was first described in a *Saccharomyces cerevisiae* model, where engineered yeast cells lacking mtDNA activated a diverse set of Rtg-dependent nuclear genes in a retrograde manner, which led to increased replicative lifespan (Kirchman *et al*. 1999). Later studies in *C. elegans* established a link between the UPR^mt^ and longevity. Early studies in 2002 found that perturbation of nuclear-encoded mitochondrial oxphos complexes, specifically complexes I, III and IV extended worm lifespan (Dillin *et al*. 2002). This was later found to be reliant on the activation of a UPR^mt^ machinery, thus suggesting that the mitohormetic benefits of mild mitochondrial perturbation was reliant on the activation of this pathway (Durieux* et al*. 2011). Moreover, glucose restriction induces formation of ROS, which increases life span through mitohormetic response in worms (Schulz* et al*. 2007). Physical exercise-induced ROS also improve insulin resistance via mitohormesis in human subjects (Ristow* et al*. 2009). Further studies in the *Drosophila* model suggested that the induction of the UPR^mt^ and insulin-like growth factor-binding proteins (IGFBP) co-regulated the longevity of flies (Owusu-Ansah *et al*. 2013). Thus, the UPR^mt^-mediated mitohormetic response is conserved from worms to humans.

Due to the longer average lifespan of mouse models, longevity studies are likely less common in mammalian models than in the lower organisms. Despite this limitation, several studies in mice have looked at longevity through the genetic ablation of *mclk1* and *Surf1* (Liu* et al*. 2005, Dell’agnello* et al*. 2007). Although the mechanism was found to be different, both mouse models showed markedly increased lifespan and increased cellular fitness. Heterozygous *mclk1* mice showed decreased activity of complex II and ROS production, which contributed to the longevity of the mice, while *Surf1* mice showed decreased mitochondrial Ca^2+^ intake and resistance to neurodegeneration. Despite the mitochondrial involvement in both models and a definite mitohormetic response, neither looked at it from the perspective of the UPR^mt^, thus necessitating further studies to establish a causal link.

One aspect of increased longevity is improved metabolic performance. Metabolism and mitochondrial integrity have been intricately linked with aging and longevity ((Lopez-Otin* et al*. 2016). In this regard, one way to study the implications of UPR^mt^ activation and its effects is through the transient modulation of systemic metabolism. A mediator of this response may be signaling factors released from tissues or organs with primary mitochondrial defects. These mediators are known as mitochondrial cytokines or mitokines and are secreted in response to mitochondrial dysfunction to re-establish systemic metabolic homeostasis. Thus, mitokine action and its improvement of metabolic parameters can be considered as a type of transient mitohormetic response and will be discussed in more detail in the following section.

### Putative mitokine factors as a regulator of systemic energy metabolism

**Figure 4:**
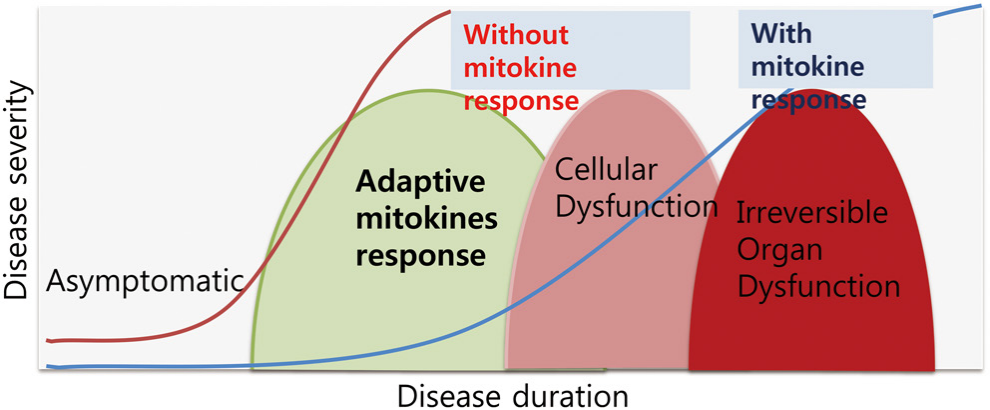
Schematic model regarding the importance of mitokine response in progression of metabolic diseases.

Previous studies in *C. elegans* have established a class of secretory proteins defined as mitochondrial cytokines or mitokines (Durieux *et al*. 2011). In this study, activation of the UPR^mt^ in the brain resulted in the distal activation of the UPR^mt^ in the worm gut and was suggested to be mediated by an unknown factor, which the authors defined as a mitokine. A subsequent study revealed that this mitokine was serotonin (Berendzen* et al*. 2016). In contrast to worm models, mitokines in the mammalian context may serve a different role. This may be explained by the increased complexity and interorgan communication distance that would be required in higher organisms, although this would require more in-depth studies to prove. Mitokine responses in mammals can be considered as critical cell non-autonomous modifiers in disease severity and progression. Secretion of mitokines can be a critical adaptive response that occurs during a specific period of disease progression (Fig. 4). Individual mitokines may act as disease markers, and utilization of these mitokine factors may serve as potential therapeutics for metabolic diseases.

Fibroblast growth factor 21 (FGF21) was reported as the first mitokine factor in mice and is induced by Atg7-knockout-mediated autophagy deficiency and mitochondrial dysfunction. Inhibition of mitochondrial function induces ATF4-dependent induction of *Fgf21* expression, leading to amelioration of diet-induced obesity and insulin resistance in mice (Kim* et al*. 2013). Currently, FGF21 is a useful serum biomarker of mitochondrial diseases, although skeletal muscle biopsy is the gold standard for diagnosis of mitochondrial translation and mtDNA maintenance disorders in humans (Suomalainen* et al*. 2011). FGF21 induced by decreased muscle fat oxidation is dependent on the AMPK and Akt1 signaling, which promotes the increase of glucose uptake in muscle and white adipose tissue browning in mice (Vandanmagsar* et al*. 2016). Moreover, FGF21 provides a thermoregulatory defense against hypothermia through activation of adipose tissue browning by enhancing adipose PGC1α (Fisher* et al*. 2012). Additionally, FGF21 attenuates diabetic cardiomyopathy by increasing AMPK activity and paraoxonase 1 signaling in streptozotocin/high-fat diet-induced hyperglycemic mice (Wu* et al*. 2017).

Emerging studies report the role of UPR^mt^ and mitohormesis on metabolic diseases across multiple species. Administration of nicotinamide riboside, a precursor of NAD^+^ biosynthesis, prevented hepatic fat accumulation in high-fat high-sucrose diet-fed mice by sirtuin-mediated UPR^mt^, triggering an adaptive mitohormesis in the liver of mice (Gariani* et al*. 2016). The UPR^mt^ induced by skeletal muscle-specific OxPhos deficiency is also associated with enhanced lipolysis and fatty acid oxidation by the induction of growth differentiation factor 15 (GDF15), thereby protecting against the adverse effects of high-fat diet-induced obesity in mice (Chung *et al*. 2017*b*). Moreover, serum GDF15 levels predict insulin resistance and glucose intolerance in nondiabetic subjects and patients with type 2 diabetes (Dostalova* et al*. 2009, Kempf* et al*. 2012, Hong* et al*. 2014). Additionally, GDF15 deficiency aggravates chronic alcohol- and carbon tetrachloride-induced liver injury by inducing infiltration of neutrophils, monocytes and activated T cells in the liver (Chung* et al*. 2017*a*). It should be noted that the relationship between the UPR^mt^ and GDF15 induction is merely correlative and requires further confirmation in follow-up studies.

Angiopoietin-like 6 is also induced by OxPhos dysfunction caused by Crif1 knockout or treatment with oligomycin and rotenone in cultured adipocytes. Angiopoietin-like 6 enhances *Fgf21* transcription via activation of ERK–MAPK pathway-mediated *Pparα* in cultured adipocytes and adipose tissue of mice (Kang* et al*. 2017). Angiopoietin-like 6 also enhances insulin sensitivity and glucose tolerance resulting from increased energy expenditure and reduction of body weight in mice (Oike* et al*. 2005).

Recently, several groups revealed that adrenomedullin 2 (ADM2), an endogenous bioactive peptide belonging to the calcitonin gene-related peptide family is an inducible gene in response to mitochondrial stress caused by inhibition of mitochondrial respiration chain in human cancer cell lines. ADM2 has a putative ATF4-binding site GTTGCATCA, located at a distance of 30 bp downstream of the translation start codon within the *ADM2* gene. Transcription of *ADM2* is tightly regulated by ATF4 during the integrated stress response, which is also associated with tumor angiogenesis by inducing vascular endothelial growth factor in the cells (Kovaleva* et al*. 2016). Moreover, ADM2 levels in plasma are negatively correlated with body weight in Chinese subjects. ADM2 overexpression in adipocytes or treatment with recombinant ADM2 improves metabolic phenotypes by inducing beiging with upregulation of UCP1, as well as M2 macrophage activation in white adipose tissues in high-fat-fed mice (Lv* et al*. 2016). Moreover, treatment with ADM2 inhibits obesity-induced insulin resistance in mice via suppression of MHCII antigen presentation in adipocytes and reduction of the activation of CD4^+^ T cells (Zhang* et al*. 2016). These data suggest that mitokines induced by mitochondrial stress may be a novel target for obesity and diabetes. Table 1 summarizes the effects of UPR^mt^ signaling components including mitochondrial chaperones, proteases and mitokines on metabolic phenotypes in mice and humans.

**Table 1:** Effects of UPR^mt^ signaling components on metabolic phenotypes in mammals.

Function	Gene name	Protein name	Metabolic phenotype	References
Chaperone	*Hspd1*	HSPD1	Reduction in hypothalamus of obese and diabetic subjects; Increase of myocardial Hspd1 in high-fructose fed rats; knockdown induces diabetes-mediated renal tubular dysfunction	Kleinridders* et al*. (2013), Aluksanasuwan* et al*. (2017)
	*Trap1*	TRAP1	Decrease of obesity and tumor formation in TRAP1 knockout	Lisanti* et al*. (2014)
	*Phb*	PHB	Overexpression in adipocytes leads to obesity in both genders, but impairment in glucose homeostasis only in male mice	Dong* et al*. (2013), Ande* et al*. (2014)
	*Hspa1a*	HSP72	Lower muscular Hsp72 expression in patients with T2DM. Overexpression enhances energy expenditure and insulin sensitivity. Knockout induces obesity and insulin resistance	Kurucz* et al*. (2002), Bruce* et al*. (2003), Drew* et al*. (2014), Henstridge* et al*. (2014)
Protease	*Oma1*	OMA1	Increased body weight and fat content, improved glucose tolerance and insulin sensitivity on NCD, but loss of metabolic benefits on HFD. Restoration of cardiac function in *Yme1l1*-deficient mice	Quiros* et al*. (2012)
	*Lonp1*	LON	Increased hepatic gluconeogenesis	Lee* et al*. (2011), Ngo* et al*. (2011)
	*Clpp*	CLPP	Amelioration of cardiomyopathy	Seiferling* et al*. (2016)
	*Yme1l1*	YME1L1	Induces dilated cardiomyopathy and heart failure	Wai* et al*. (2015)
	*Parl*	PARL	Impaired insulin signaling in skeletal muscle	Walder* et al*. (2005), Civitarese* et al*. (2010)
Mitokine	*Gdf15*	GDF15	GDF15 levels in serum increase in patients with T2DM. Associated with fatty acid oxidation and lipolysis. Reduces liver injury by modulating immune cell infiltration	Dostalova* et al*. (2009), Kempf* et al*. (2012), Hong* et al*. (2014), Chung* et al*. (2017*a* ,*b*)
	*Adm2*	ADM2	Negative correlation with body weight. Overexpression in adipocytes induces beiging of white adipose tissue as well as M2 polarization of adipose macrophages. Recombinant ADM2 inhibits insulin resistance by deactivating adipose CD4+ T cells	Lv* et al*. (2016), Zhang* et al*. (2016)
	*Angptl6*	ANGPTL6	Required for Fgf21 expression in adipocytes. Increases insulin sensitivity, glucose tolerance and energy expenditure	Oike* et al*. (2005), Kang* et al*. (2017)
	*Fgf21*	FGF21	Increases fat oxidation and mitochondrial function. Enhances glucose uptake in muscle and browning in white adipose tissue. Attenuates diabetic cardiomyopathy	Fisher* et al*. (2012), Kim* et al*. (2013), Vandanmagsar* et al*. (2016), Wu* et al*. (2017)

HFD, high-fat diet; NCD, normal chow diet; T2DM, type 2 diabetes mellitus.

Lastly, mitochondrial-derived peptides also have a key role in stress resistance as a mitochondrial autocrine, paracrine and endocrine signal. Humanin, which is translated in the mitochondria (21-amino-acid peptide) or the cytoplasm (24-amino-acid peptide), has been shown to suppress neuronal cell death caused by Alzheimer’s disease and to increase cytoprotective effects against stress and disease models (Lee* et al*. 2013). MOTS-c is also a mitochondrial-derived peptide that improves obesity and insulin resistance in mice (Lee* et al*. 2015), but the mechanism regarding translation of MOTS-c is not fully understood and requires further studies for understanding the natural course and tissue specificity of MOTS-c expression, as well as the metabolic effects of MOTS-c in humans.

### Induction of UPR^mt^ in physiological and pharmacological models

The UPR^mt^ can be activated in mammals as well as worms by physiological and pharmacological perturbations. Nuclear transcriptional response is induced when the mis-or unfolded proteins within the mitochondrial matrix accumulate by a variety of mitochondrial stresses. In worms, activation of UPR^mt^ requires the activating transcription factor associated with stress-1 (ATFS-1), which is essential for regulating an important transcriptional response to recover mitochondrial function (Nargund *et al*. 2015). The degraded peptides in matrix by mitochondrial proteases were exported into the cytosol via HAF-1, an ATP-binding cassette transporter protein (Haynes *et al*. 2010). HAF-1 also modulates ATFS-1-mediated UPR^mt^ activation by inhibiting mitochondrial import of ATFS-1 (Nargund* et al*. 2012). ATFS-1 was also involved in the maintenance and propagation of deleterious mtDNA in a heteroplasmic *C. elegans* strain (Lin* et al*. 2016). In mammals, the UPR^mt^ and mitokine induced by mitochondrial dysfunction requires CHOP-mediated p38 kinase activation in skeletal muscle (Chung *et al*. 2017*b*). This study also revealed putative CHOP-responsive elements in the human promoter of GDF15, a putative mitokine factor (Chung *et al*. 2017*b*). The UPR^mt^ response element was also identified within the promoters of both CHOP and C/EBPβ genes in mammalian cells (Horibe & Hoogenraad 2007). Moreover, activating transcript factor 5 (ATF5), one of the bZIP transcription factors, regulates the activation of UPR^mt^ by a stress-dependent shift in its cellular localization, thereby promoting proliferation and recovery from mitochondrial stress in mammalian cells (Fiorese *et al*. 2016). These data suggest that UPR^mt^ is an evolutionally conserved mechanism from worms to mammals.

Pharmacological induction of UPR^mt^ is also extensively characterized in mammalian cells. Treatment with doxycycline induces mitonuclear protein imbalance in cultured cells as well as in tissues from mice, thereby triggering the UPR^mt^ to recover the proper mitochondrial function (Moullan* et al*. 2015). Gamitrinib-triphenylphosphnium (GTPP), a specific inhibitor of the mitochondrial matrix heat shock protein 90 (HSP90), induces the transcription of *HSPD1* and *HSPE1* as markers of UPR^mt^ in HeLa cells (Munch & Harper 2016). GTPP also promotes the accumulation of misfolded proteins in the mitochondria, thereby activating SIRT3 and its downstream target genes (Papa & Germain 2014). Actinonin generates an aberrant accumulation of *de novo* mitochondrial proteins in the inner membrane and impairs the turnover of *de novo* mitochondrial protein synthesis (Richter* et al*. 2015). MitoBloCK compounds (Mitochondrial protein import Blockers from the Carla Koehler lab), a selective inhibitor of the sulfhydryl oxidase Erv1 activity, regulates the translocation of redox-regulated proteins into mitochondria and oxidation of Tim13 and Cmc1 (Dabir* et al*. 2013). Moreover, 2-cyano-3,12-dioxo-oleana-1,9(11)-dien-28-oic acid (CDDO), an inhibitor of the LON matrix protease, triggers the robust upregulation of mitochondrial chaperones and proteases in C2C12 myotubes (Chung *et al*. 2017*b*).

Although various UPR^mt^ inducers have been used in experimental studies, these compounds are not free from the issues of mitochondrial specificity or poor property. However, a variety of studies showing the physiological and pharmacological activation of UPR^mt^ signaling demonstrates the cell autonomous footprint of the UPR^mt^, which leads to beneficial phenotypic responses such as improved metabolic fitness and longevity in worms and flies. Moreover, genetic or pharmacologic modulation of OxPhos system regulates metabolic phenotypes across the animal kingdom through induction of UPR^mt^ and mitohormesis (Durieux *et al*. 2011, Shao* et al*. 2016, Chung *et al*. 2017*b*). Thus, the utilization of these pharmacological UPR^mt^ inducers can facilitate further studies on the effects of UPR^mt^ and its physiological relevance, including metabolic effects.

## UPR^mt^ and mitohormesis as new therapeutic targets for metabolic diseases

What is the implication of UPR^mt^ and mitohormesis for the development of metabolic diseases? Indeed, a modest or transient mitochondrial stress provides fundamental protection to the host through cell autonomous and cell-non-autonomous signaling, leading to prevention of metabolic diseases and aging-related disorders. The protective function of UPR^mt^ and mitohormesis in multiple organisms, including accumulation of deleterious mitochondrial genomes, pathogen infection, hematopoietic stem cell maintenance and aging has been established (Lin & Haynes 2016, Qureshi* et al*. 2017). The UPR^mt^-mediated metabolic adaptation may serve to rewire cellular metabolism to recover or prevent the metabolic deterioration in humans.

Recently, we revealed that OxPhos deficiency in skeletal muscle (Chung *et al*. 2017*a*) activated UPR^mt^ and the induction of mitokines, thereby maintaining systemic energy homeostasis in metabolic deterioration during diet-induced obesity. Additionally, FGF21, a starvation-induced hormone, is a well-known mitokine that elicits metabolic benefits by increasing fat oxidation and improving mitochondrial function in obese subjects (Kim *et al*. 2013, Wall* et al*. 2015). In addition to its physiologic actions, the pharmacologic functions of FGF21 play a key role in the maintenance of systemic energy balance through modulating feeding behavior and energy expenditure (Kim & Lee 2014, Laeger* et al*. 2014). As discussed earlier, the mitokines such as angiopoietin-like 6 and ADM2 may also have the therapeutic value in the treatment of obesity and insulin resistance in humans. Despite these studies, the relevance of the UPR^mt^ and mitohormesis is only beginning to emerge and it is still unknown if the mitonuclear communication network and cell non-autonomous hormonal factors exert beneficial effects in human metabolic diseases.

## Conclusions and perspectives

Metabolic diseases, including diabetes, obesity and cardiovascular diseases, which are strongly associated with mitochondrial dysfunction, have become a global epidemic and is a major worldwide health concern. The growing prevalence of metabolic diseases induced by genetic and environmental factors increases annual direct medical costs in the world. Mitochondrial research has been mainly focused on the association between OxPhos and metabolic diseases as well as basic structural principles of OxPhos. Until recently, the molecular and holistic approaches toward mitochondrial proteostasis were merely developed in the physiologic and pathologic contexts from yeast to mammals. In the near future, the UPR^mt^ may be an attractive therapeutic target for the treatment of metabolic diseases that result from mitochondrial dysfunction. Chemical and pharmacologic agents that target mitochondrial oxidative phosphorylation and proteostasis should be developed and investigated in the basic and clinical fields.

We have highlighted the relationship between mitochondrial OxPhos and metabolic diseases in multiple organisms where the UPR^mt^ and mitohormesis have been demonstrated to play a protective role in obesity and diabetes. However, important questions remain unanswered about the UPR^mt^ and mitohormesis in the development and maintenance of metabolic diseases. The most important question would be how UPR^mt^ regulates the susceptibility of metabolic diseases in mammals. It would also be interesting to determine how mitohormesis affects systemic energy metabolism, leading to inhibition of obesity and insulin resistance.

Although the physiologic roles of the UPR^mt^ and mitohormesis in adapting metabolism to recover mitochondrial proteostasis and energy homeostasis are beginning to emerge, the data provide convincing evidences to suggest that UPR^mt^-mediated cellular or systemic protective effects may be beneficial for the development of therapeutics for diverse metabolic diseases such as diabetes, neurodegenerative diseases and aging-related disorders. However, it should be noted that while the association between UPR^mt^/mitohormesis and metabolic diseases is well characterized from worms to flies, less is known regarding the mechanism of the UPR^mt^ and the receptors for mitokines in mammals. It is reasonable to predict that mitokines may have autocrine, paracrine and endocrine effects on other cell types within the tissue. Furthermore, mitokine receptors may be present in distant organs, suggesting that mitokines play endocrine roles in interorgan communication involved in systemic homeostasis.

## Declaration of interest

The authors declare that there is no conflict of interest that could be perceived as prejudicing the impartiality of this review.

## Funding

This work was supported by Global Research Laboratory (GRL) Program, National Research Foundation of Korea, Ministry of Science and ICT (2017K1A1A2013124, 2017R1E1A1A01075126), as well as by a grant (H S Y, 2017F-1) from the Korean Diabetes Association. H S Y was also supported by the Basic Science Research Program, National Research Foundation, Ministry of Science and ICT, Future Planning, Korea (2015R1C1A1A01052432, NRF-2018R1C1B6004439). J Y C was supported by the Basic Science Research Program through the NRF funded by the Ministry of Science, ICT, and Future Planning, Korea (NRF-2014M3A9D8034464).

## Acknowledgements

The authors apologize in advance for the authors who have been unintentionally left out in this review.
